# Outbreak of Foodborne Botulism Associated with Improperly Earthenware Cheese (Koupé): A Case Report

**Published:** 2019-10

**Authors:** Parshang FAGHIH SOLAYMANI, Ardeshir RAHIMZADEH, Farideh MOSTAFAVI

**Affiliations:** Communicable Diseases Control Group, Kurdistan University of Medical Sciences, Sanandaj, Iran

**Keywords:** Foodborne botulism, Outbreak, Cheese, Iran

## Abstract

Here we report the consumption of traditional cottage cheese (Koupé) in western Iran, as a new way of transmission of botulism. All the patients (a nine member family) had at least two specific symptoms of botulism. Given the clinical symptoms and contact history, antitoxins were injected in the early hours of hospital admission. On Jan 27, 2017, three patients clinically suspected of foodborne botulism were referred to the hospital in Sanandaj, western Iran from their local hospital in Baneh, western, Iran. Because of the worsening of clinical conditions, a 34-yr-old man with both gastrointestinal and neurological symptoms was admitted to the ICU, while other family members were treated in the infectious diseases ward of the hospital. The disease was diagnosed through isolating toxin A from the cheese and testing the serum sample of one of the patients. This case of botulism showed that traditional Koupé cheese could cause foodborne botulism. Hence, it is necessary to train and inform people about how to process and keep the cheese to prevent similar cases.

## Introduction

*Clostridium botulinum* is an anaerobic, spore-forming, gram-positive bacillus naturally found in soil and sediments around the world (1). There are seven serotypes of *C. botulinum* named A-G, among them only A, B, E, and F cause disease in humans (2, 3).

In general, the symptoms of botulism poisoning are abdominal cramps, double or blurred vision, shortness of breath, muscle weakness, and other serious symptoms such as nausea, vomiting, fatigue, dizziness, dry skin, dry mouth and throat, ptosis, swallowing problems, body aches, and paralysis (4).

Foodborne botulism is caused due to the consumption of contaminated home-canned foods and vegetables, traditional seafood (such as fermented fish, smoked fish), and improperly preserved foods; it also occasionally emerges as the result of eating unpasteurized dairy products (6). This is the first reported case of botulism outbreak associated with the consumption of Koupé (jug or pot cheese) in the world.

## Case Report

On Jan 27, 2017, three patients clinically suspected of foodborne botulism were referred to the hospital in Sanandaj, western Iran from their local hospital in Baneh, western Iran. They had suffered from gastrointestinal symptoms for three days before hospital admission. Learning that the patients had eaten a common meal, the clinicians reported botulism as the likely diagnosis and immediately started the botulism management procedure and requested botulinum antitoxin ABE treatment.

One of the cases (a 34-yr-old man) with both gastrointestinal and neurological symptoms such as dyspnea, sore throat, dysphasia, severe ptosis, diplopia, and muscle weakness, while he was conscious, was transferred to intensive care unit. The patient's vital signs were T (temperature) =37.4, r (respiratory) =20, Bp (Blood pressure) =130/80 and p (pulse) =96 respectively. Heart and lung examination was normal. There was no abdominal tenderness and dilation. In gag reflex assessment, he had dysarthria. Force muscle score was normal but he had muscle weakness. In the second day, he still had swallowing problems.

The experiments were performed on Alt, Ast, Ak.p, ESR, U/A, INR, WBC, CBC, cpk, na, k, BUN, cr, BS, PT, PTT, with abnormal WBC=11.8 and cpk=408. After two days, he was transferred to infectious ward. In the 8th day, he had still difficulty in gag reflex but he was able to drink liquids. Prescribed drugs included ranitidine, enoxaparin sodium, intralipid and amino plasma.

The next day (Jan 28, 2017), three clinically suspected cases were admitted to the same hospital with gastrointestinal symptoms onset within the same time span as the previous cases were reported. At the same time, three clinically suspected cases were admitted to the local hospital in Baneh City (Table 1).

**Table 1:** Demographics characteristics of the patients infected with foodborne botulism

***Patient***	***Sex***	***Age (yr)***	***Weight (kg)***	***Height (cm)***	***Smoking***	***Blood pressure***	***Body Mass Index (BMI)***
1	Male	28	75	170	No	100/60	24.5
2	Male	34	73.5	171	Yes	130/80	25.1
3	Male	31	80	168	Yes	130/80	28.3
4	Male	35	75. 5	173	No	110/70	25.2
5	Female	32	62	65	No	120/80	22.8
6	Male	52	63	165	Yes	140/100	23.1
7	Male	61	75	173	No	120/80	25
8	Female	7	24.5	103	No	100/70	23.1
9	Female	9	22	101	No	100/70	21.5

The duration of hospitalization of the patients varied from 8 to 12 d. The patients almost recovered and had no neurological or gastrointestinal symptoms were discharged from the hospital after obtaining personal consent and a specialist weekly for a month followed them (Table 2).

Clinical specimens obtained from all the patients and the samples of suspected food (jug or pot cheeses) were sent to the Pasteur Laboratory in two consecutive days. The results were obtained by phone two days later. Finally, serological tests confirmed the presence of botulinum toxin type A in the blood of only one of the cases and in jug cheese as well.

**Table 2:** Clinical finding of cases infected with foodborne botulism

***Patient***	***Signs and symptoms***	***Botulinum toxin***	***Serum sampling date***	***Admission date***	***Duration of hospitalization (days)***	***Koupe consumption***	***Disease history***	***Specialist visit***
1	Muscle weakness, dysphagia	Negative	2017 /28/ 1	2017 /28/ 1	8	Positive	-	Neurologist visit & Infectious specialist
2	Dyspnea, sore throat, dysphasia, severe ptosis, diplopia and muscle weakness	Positive	2017 /27/ 1	2017 /27/ 1	12	Positive	-	Neurologist & Infectious specialist
3	Neck muscle weakness, pain in the back of neck, nausea	Negative	2017 /28/ 1	2017 /28/ 1	9	Positive	-	Infectious specialist
4	Dysphagia, neck muscle weakness, ptosis, neck pain, dysphagia	Negative	2017 /28/ 1	2017 /28/ 1	8	Positive	-	Infectious specialist
5	Ptosis, lower limb weakness, nausea	Negative	2017 /28/ 1	2017 /27/ 1	12	Positive	Migraine	Infectious specialist
6	Neck muscle weakness	Negative	2017 /28/ 1	2017 /28/ 1	9	Positive	-	Infectious specialist
7	Neck muscle weakness, pain in the back of neck	Negative	2017 /28/ 1	2017 /28/ 1	8	Positive	-	Infectious specialist
8	Muscle weakness, dysphagia	Negative	2017 /28/ 1	2017 /27/ 1	8	Positive	-	Infectious specialist
9	Neck muscle weakness, pain in the back of the neck	Negative	2017 /28/ 1	2017 /28/ 1	9	Positive	-	Infectious specialist

All the cases were from Baneh City, Kurdistan Province, in west of Iran. Data were collected from hospitals and coordinating with Vice Chancellor for Clinical Affairs, Kurdistan University of Medical Sciences, Sanandaj, Iran. This study was conducted according to the guidelines laid down in the Declaration of Helsinki and Informed consent was obtained from all participants.

## Discussion

Foodborne botulism occurs due to a failure in processing canned food especially home canning or home processing of food (6). Foodborne botulism is an important disease in Asia because many people use fermented foods, for instance, 86% of foodborne botulism outbreaks are reported in Japan between 1995 and 1998 (7).

The first foodborne botulism outbreak in Iran occurred in Rasht in 1966 and at least 28 patients with botulism were hospitalized (8).

This is the first reported case of botulism outbreak caused by jug or pot cheeses in the world. Koupe is a kind of the earthenware cheese product called Koupe in the northwest of the country. Koupe is traditionally produced from sheep or cow milk. It is largely produced in Greece, Turkey (Carra, Coekelek, and Otlu peynir), and Iran (Koupe) (9). To produce this type of cheese, first the raw milk is heated up to 40 °C and cooled to 32 °C, then curd is added and they are mixed until proper coagulation, finally, it is cut into pieces and pressed for an hour to drain. The obtained pieces are transferred to saturated saltwater (20%) and kept for 24 h in 20 °C. After pouring salt on both sides of the cheeses for three days, it is kept in saltwater (pour) for 2 months in a refrigerator (5–8 °C). Finally, the cheese is placed in pottery or other earthenware containers and buried 1.5 m underground for 3 to 6 months in anaerobic conditions. Today, some cheese producers use a plastic cofferdam or metal container instead of a jar (9).

Of all the blood samples taken from the nine patients, only the blood serum of one transferred to intensive care unit was positive and the rest were negative; it may be attributed to the exposure of the patient to a higher level of toxins or bacteria, or receiving antitoxin before testing.

One limitation of this study is recall bias, questions related to the consumption foods and how much were exactly eaten.

## Conclusion

This study is the first to report this foodborne botulism occurs due to processing and storing this type of cheeses. Considering this procedure of cheese processing and storing can be dangerous; public health officials should be immediately informed of clinical diagnosis and report similar cases as soon as possible. The health authorities must collaborate in educating people about disadvantages of processing and storing this type of cheeses to prevent additional cases. Manufacturers that produce Koupe for commercial use must ensure their compliance with food safety regulations.

## Ethical considerations

Ethical issues (Including plagiarism, informed consent, misconduct, data fabrication and/or falsification, double publication and/or submission, redundancy, etc.) have been completely observed by the authors.
